# Draft Genome Sequences of Clinical Daptomycin-Nonsusceptible Methicillin-Resistant *Staphylococcus aureus* Strain APS211 and Its Daptomycin-Susceptible Progenitor APS210

**DOI:** 10.1128/genomeA.00568-15

**Published:** 2015-06-11

**Authors:** David R. Cameron, Jhih-Hang Jiang, Iain J. Abbott, Denis W. Spelman, Anton Y. Peleg

**Affiliations:** a Department of Microbiology, Monash University, Melbourne, Australia; b Department of Infectious Diseases, The Alfred Hospital and Monash University, Melbourne, Australia; c Department of Microbiology, The Alfred Hospital, Melbourne, Australia

## Abstract

To assess the genetic factors contributing to daptomycin resistance in *Staphylococcus aureus*, the draft genome of a clinically derived daptomycin-nonsusceptible isolate APS211 was generated and compared to the draft sequence of its susceptible progenitor strain APS210. Four genetic differences were identified including a previously described mutation within the *mprF* gene.

## GENOME ANNOUNCEMENT

Daptomycin is a cyclic lipopeptide antibiotic that is increasingly relied upon for the treatment of methicillin-resistant *Staphylococcus aureus* (MRSA) infections (1). Daptomycin-nonsusceptible strains (defined by a MIC > 1.0 µg/mL) have recently been described, however, the genetic mechanism behind the emergence of these strains is not completely understood (2, 3). We, and others, have performed whole-genome sequencing of daptomycin-exposed strains, identifying point mutations in numerous genes including those important for phospholipid metabolism (most notably the lysl-phosphatidylglycerol synthase, encoded by *mprF*), as well as the two-component regulatory system, *walKR,* and RNA polymerase subunits *rpoB* and *rpoC* (2, 4–6). In order to further understand the development of daptomycin resistance in *S. aureus*, we prepared the draft genome sequence of a daptomycin-susceptible strain, APS210 (daptomycin MIC 0.5 µg/mL), isolated from a patient with bacteremia and compared it to its daptomycin nonsusceptible derivative, APS211 (daptomycin MIC 4.0 µg/mL), that emerged after daptomycin treatment and therapeutic failure.

Genomic DNA from APS210 and APS211 was prepared using a QIAGEN blood and tissue kit per the manufacturer’s instructions and sequenced using Illumina MiSeq (250-bp paired-end reads) generating 5,255,342 and 4,468,075 reads, respectively. Draft genomes were assembled *de novo* using CLC workbench (v6.0.2) with >1,000× coverage and each genome was annotated using the NCBI Prokaryotic Genomes Annotation Pipeline. The genome sequence of APS210 comprised 83 contigs (>500 bp in length, *N*_50_ = 74,505 bp) with a combined length of 2,906,880 bp, G+C content of 32.7%, and 2,859 predicted protein-coding regions. The sequence of APS211 comprised 72 contigs (>500 bp in length, *N*_50_ = 73,502 bp) with a combined length of 2,905,661 bp and G+C content of 32.7%. Both strains were sequence type (ST) 45 and staphylococcal cassette chromosome *mec* (SCC*mec*) type V (7). Comparison of the two genomes revealed four single nucleotide polymorphisms (SNPs) including a mutation that led to the previously described MprF amino acid substitution, S337L (2, 8). Two of the SNPs were predicted to be synonymous within hypothetical proteins (a predicted phage protein and a protein with similarity to *N*-acyl-l-amino acid amidohydrolase) and the final SNP was predicted to be intergenic. Defining the precise molecular mechanisms behind MprF-mediated daptomycin-nonsusceptibility in *S. aureus* is ongoing in our laboratory.

### Nucleotide sequence accession numbers.

The whole-genome shotgun sequences of APS210 and APS211 have been deposited in DDBJ/EMBL/GenBank under the accession numbers JXUD00000000 and JXUV00000000, respectively.
